# Cellular Localization of Wheat High Molecular Weight Glutenin Subunits in Transgenic Rice Grain

**DOI:** 10.3390/ijms18112458

**Published:** 2017-11-18

**Authors:** Yeong-Min Jo, Kyoungwon Cho, Hye-Jung Lee, Sun-Hyung Lim, Jin Sun Kim, Young-Mi Kim, Jong-Yeol Lee

**Affiliations:** 1 National Institute of Agricultural Science, Rural Development Administration, Jeonju 54874, Korea; whdudalsv@naver.com (Y.-M.J.); kw.cho253@gmail.com (K.C.); leehx@korea.ac.kr (H.-J.L.); limsh2@korea.kr (S.-H.L.); jskim220@korea.kr (J.S.K.); 2 Department of Biotechnology, College of Agriculture and Life Sciences, Chonnam National University, Gwangju 61186, Korea; 3 Division of Life Science, College of Life Sciences and Biotechnology, Korea University, Seoul 02841, Korea

**Keywords:** seed storage proteins, high-molecular-weight glutenin subunits, *1Dx5_KK*, *1Dy10_JK*, glutelin, prolamin

## Abstract

Rice (*Oryza sativa* L.) is a primary global food cereal. However, when compared to wheat, rice has poor food processing qualities. Dough that is made from rice flour has low viscoelasticity because rice seed lacks storage proteins that are comparable to gluten protein from wheat. Thus, current research efforts aim to improve rice flour processing qualities through the transgenic expression of viscoelastic proteins in rice seeds. In this study, we characterized the transgenic expression of wheat glutenin subunits in rice seeds. The two genes *1Dx5_KK* and *1Dy10_JK*, which both encode wheat high-molecular-weight glutenin subunits that confer high dough elasticity, were cloned from Korean wheat cultivars KeumKang and JoKyung, respectively. These genes were inserted into binary vectors under the control of the rice endosperm-specific *Glu-B1* promoter and were expressed in the high-amylose Korean rice cultivar Koami (*Oryza sativa* L.). Individual expression of both glutenin subunits was confirmed by SDS-PAGE and immunoblot analyses performed using T_3_ generation of transgenic rice seeds. The subcellular localization of 1Dx5_KK and 1Dy10_JK in the rice seed endosperm was confirmed by immunofluorescence analysis, indicating that the wheat glutenin subunits accumulate in protein body-II and novel protein body types in the rice seed. These results contribute to our understanding of engineered seed storage proteins in rice.

## 1. Introduction

Seed storage proteins (SSPs) in cereal grains serve as a source of nitrogen during germination and play important roles in food processing [1]. SSPs accumulate predominantly in sub-aleurone and aleurone layers of the seed endosperm. In rice, glutelins account for 60–80% of total SSPs, whereas the major SSPs in other cereals are prolamins, including hordeins in barley, secalins in rye, avenins in oats, and gluten proteins that are composed of gliadin and glutenin subunits in wheat [2]. These prolamins are responsible for the elastic properties of dough made from these cereals. 

In wheat (*Triticum aestivum* L.), SSPs are categorized into gluten and non-gluten families. Gluten, formed via cross-links between monomeric gliadins and multimeric glutenins, is a protein complex that confers dough viscosity and extensibility [2]. The glutenins are classified as either low- or high-molecular-weight glutenin subunits (LMW-GSs and HMW-GSs, respectively). HMW-GSs are a crucial factor in dough strength and quality [3]. The HMW-GSs in hexaploid bread wheat are encoded by *Glu-1* loci (*Glu-A1*, *Glu-B1*, and *Glu-D1*) on the long arm of group 1 chromosomes. Each locus encodes two different subunits, with HMW and LMW subunits denoted as x- and y-type subunits, respectively. Theoretically, there are six possible HMW-GS types in hexaploid bread wheat cultivars, including 1Ax, 1Ay, 1Bx, 1By, 1Dx, and 1Dy [2,3,4]. However, due to the silencing of several HMW-GSs, including 1Ay, following wheat domestication, HMW-GSs are present in various combinations of three to five types in individual bread wheat cultivars [2,5]. Recent studies have shown that allelic forms of HMW-GSs that are present in bread wheat cultivars contribute towards bread-making quality, and thus they are assigned a quality score from one to five that facilitates the prediction of a cultivar’s bread-making suitability. Based on these quality scores, the 1Dx5 glutenin subunit has a large positive effect on wheat dough elasticity [2,5]. Wheat glutenins are synthesized in the endoplasmic reticulum (ER), which are assembled via ER chaperones, and then transported to protein storage vacuoles (PSVs) either directly or via the Golgi apparatus [6,7,8].

In rice (*Oryza sativa*), glutelins (encoded by 15 genes), prolamins (34 genes), and a globulin (one gene) account for 60–80%, 20–30%, and 2–8% of the total SSP, respectively [9,10,11]. The rice glutelins are composed of acidic (30–39 kDa) and basic (19–25 kDa) subunits. The prolamins are classified as 10-, 13-, or 16-kDa prolamins, depending on their mobility in SDS-PAGE. Furthermore, the 13-kDa prolamins form two subgroups that are based on cysteine residue number, including high-sulfur prolamin 13a and low-sulfur prolamin 13b [10,12,13,14]. Rice prolamins are comparable to prolamins of other cereals, such as γ-hordein in barley, α-/β- and γ-gliadins in wheat, and γ-secalin in rye [15,16,17]. They have a high level of glutamine, low levels of lysine, histidine, cysteine, and methionine [18], and confer less elasticity and cohesive properties when compared to wheat glutenins [19]. Like wheat SSPs, rice glutelins and prolamins are synthesized in the rough endoplasmic reticulum (ER) and are assembled in the ER lumen via ER chaperones. After that, however, rice glutelins are deposited into PSVs (protein body-II, PB-II) via the Golgi apparatus, whereas rice prolamins form ER-derived spherical protein bodies (protein body-I, PB-I) [1,6,20,21].

Efforts to improve the properties of rice flour for food processing are relevant in many countries where rice is a staple food. Genetic engineering approaches have been adopted to improve the nutritional and processing properties of rice, as well as in efforts to produce recombinant pharmaceutical proteins in rice. For example, soybean β-conglycinin subunits were expressed in rice seeds to improve their nutritional and physiological properties, which resulted in the accumulation of PB-II that formed a complex with rice glutelin via disulfide bonds [22]. Moreover, Takagi et al. [23] reported that PB-I-localized 3Crp (three T-cell epitopes derived from cedar pollen allergens) is more tolerant of enzymatic digestion in the gastrointestinal tract when compared to the chemically synthesized and PB-II-localized forms, suggesting that rice seed PB-I may be appropriate for edible vaccine delivery. These results also indicate that it is important to understand where in the cell such exogenous proteins accumulated.

The addition of wheat gluten during the production of dough from rice improves the quality of the resulting rice bread [24,25]. In addition, the over-expression of wheat 1Dx5 and/or 1Dy10 HMW-GSs improves the extensibility and elasticity of dough from wheat [26,27,28] and rye [29]. In efforts that were aimed at improving rice dough quality, preliminary attempts have been made to express wheat SSPs, including 1Ax1 [30], 1Bx7 [31], 1Dx5 [32,33], and 1Dy10 [34] in rice seeds, although to date, only 1Dx5 has been shown to confer a positive effect on rice dough properties. The objective of this study was to engineer wheat HMW-GS into rice seeds. Representative glutenin genes with high elasticity and viscosity properties, *Dx5* and *Dy10*, were cloned from Korean wheat cultivars KeumKang and JoKyung, herein referred to as *1Dx5_KK* and *1Dy10_JK*. These genes were inserted into expression cassettes and were then expressed in the high-amylose Korean rice cultivar Koami (*Oryza sativa* L.) under the control of the rice endosperm-specific *Glu-B1* promoter [35]. Individual transgenic rice lines expressing 1Dx5 and 1Dy10 were characterized by SDS-PAGE and immunoblot analysis, and the cellular localization of 1Dx5 and 1Dy10 in rice seed endosperm was observed by immunofluorescence. Our results showed that the wheat glutenin subunits accumulate in the endosperm of transgenic rice seeds, predominantly in the aleurone layer versus the starchy endosperm, and at the subcellular level are deposited into protein body-II and novel protein body types in the rice seed.

## 2. Results and Discussion

### 2.1. High-Molecular-Weight Glutenin Subunits (HMW-GSs) Amino Acid Sequence Analysis 

**Figure 1:**
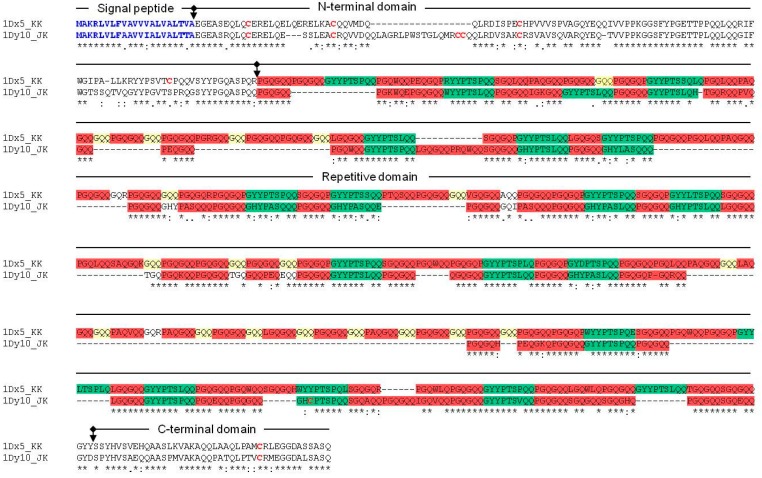
Amino acid sequences and primary structures of 1Dx5_KK and 1Dy10_JK. Each domain is separated with arrow. Yellow background, x-type specific GQQ repetitive peptides; green background, GYYPTSP(L)QQ; red background, PGQGQQ; red letters, cysteine residues; blue letters, signal peptides.

Previously, we performed allelic analysis of HMW-GSs among Korean wheat cultivars and evaluated their food processing qualities [36]. This led to the identification of two HMW-GSs with high scores, specifically 1Dx5_KK and 1Dy10_JK. Sequence analysis shows that these proteins consist of a signal peptide, an N-terminal domain, a repetitive domain, and a C-terminal domain (Figure 1). 1Dx5_KK has five cysteine residues, with four and one present in the N-terminal and C-terminal domain, respectively, whereas 1Dy10_JK has seven cysteine residues, with five, one, and one that were present in the N-terminal, repetitive, and C-terminal domains, respectively. The majority of x- and y-type subunits in HMW-GSs typically have four and seven cysteine residues, respectively. Cysteine residue number is believed to be associated with bread quality [37], indicating that one extra cysteine residue in the N-terminal domain of Dx5_KK could affect subsequent food-processing qualities. Indeed, according to the elastic and viscous properties of glutenin, the *Glu-1* loci are ranked as *Glu-D1* > *Glu-B1* > *Glu-A1* and allelic HMW-GSs at *Glu-D1* loci are ranked as Dx5 + Dy10 > Dx2 + Dy12 > Dx4 + Dy12 [37,38].

The x- and y-type subunits of wheat HMW-GSs share the same structure as 1Dx5_KK and 1Dy10_JK in that they contain N-terminal, repetitive, and C-terminal regions. A unique tri-peptide motif (GQQ) is present in the x-type subunit, whereas in y-type subunit, the second proline in the GYYPTSPQQ motif is replaced by a leucine (GYYPTSLQQ). Our 1Dx5_KK and 1Dy10_JK sequence analysis showed that the GQQ motif (yellow in Figure 1) is present only in 1Dx5_KK, and the GYYPTSPQQ motif and the modified version GYYPTSLQQ (Green in Figure 1) are repeated thirteen and three times in 1Dx5_KK, and four and nine times in 1Dy10_JK, respectively (Figure 1). 

Choi et al. [39] reported that the signal peptides of prolamin in rice and zein in maize share high homology, and direct proteins to PB-I derived from ER lumen. Comparable to the high homology between signal peptides of HMW-GSs [4], there is high homology between 1Dx5_KK and 1Dy10_JK signal peptides, suggesting that these proteins could be targeted to the same organelle. However, the homology between rice prolamin and wheat HMW-GS is not high, indicating that subcellular targeting of the wheat HMW-GSs 1Dx5_KK and 1Dy10_JK in transgenic rice could be different to that of rice prolamin.

### 2.2. Selection of Transgenic Rice Plants Expressing Wheat HMW-GSs

**Figure 2:**
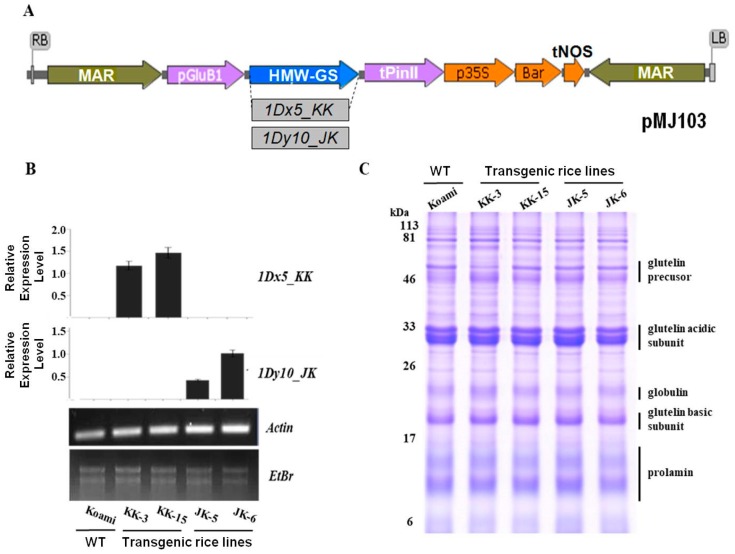
Binary vector construction and generation of transgenic rice plants expressing wheat high-molecular-weight glutenin subunits (HMW-GSs) 1Dx5_KK and 1Dy10_JK. (**A**) Binary vector used to generate transgenic rice plants expressing wheat HMW-GSs 1Dx5_KK and 1Dy10_JK. The pMJ vector has a *Glu-B1* promoter, *Bialaphos resistance* (*Bar*) gene as herbicide-resistance marker, and *Nopaline synthase* terminator (*tNOS*); (**B**) Transcript level analysis of *1Dx5_KK* and *1Dy10_JK* genes in seeds of independent KK and JK transgenic rice lines, respectively, using quantitative real-time PCR; and, (**C**) Expression analysis of rice total seed storage proteins (SSPs) in mature seeds of Koami (wild-type) and independent transgenic rice lines. Total SSPs (10 μg) were separated using SDS-PAGE (12.5%) and visualized using Coomassie brilliant blue (CBB) staining. WT, wild type; KK and JK, transgenic lines expressing 1Dx5_KK and 1Dy10_JK, respectively.

To create transgenic rice plants overexpressing wheat HMW-GSs, binary vectors were constructed, as shown in Figure 2A, with the expression of *1Dx5_JK* and *1Dy10_KK* under control of the rice endosperm-specific *Glu-B1* promoter. The transgenes were inserted into the genome of *japonica*-type Korean rice cultivar (cv.) Koami using Agrobacterium-mediated transformation, resulting in the isolation of 13 and 6 independent T_0_ transgenic rice lines overexpressing 1Dx5_KK and 1Dy10_JK in rice seeds, respectively (Figure S1). Among the transgenic rice lines that survived to the next generation (T_1_), two representative homozygous lines, namely KK-3 and KK-15 lines, expressing 1Dx5_KK, and JK-5 and JK-6 lines expressing 1Dy10_JK, were selected from the progeny (T_4_ generation) of the transgenic lines based on qRT-PCR analysis (Figure 2B).

**Figure 3:**
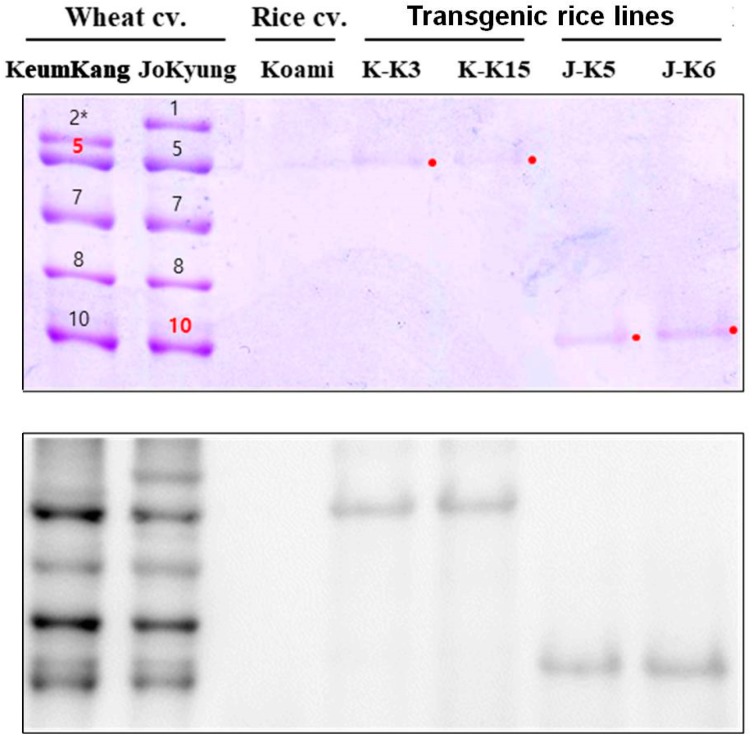
SDS-PAGE (12.5%) and immunoblot analyses of *1Dx5_KK* and *1Dy10_JK* expression in mature seeds of wheat cultivars KeumKang and JoKyung, rice cultivar Koami (wild-type), and KK and JK independent transgenic rice lines expressing *1Dx5_KK* and *1Dy10_JK*, respectively. Membranes were hybridized with HMW-GS polyclonal antibodies. Number 1, 2*, 5, 7, 8, 10 in wheat cultivar (cv.) KeumKang and JoKyung indicate the allelic composition of glutelin as 1Ax1, 1Ax2*, 1Dx5 (upper red number and point), 1Bx7, 1By8, and 1Dy10 (lower red number and point).

In the seeds of the T_4_ transgenic rice lines, no 1Dx5_KK or 1Dy10_JK expression or differential expression of SSPs was observed following SDS-PAGE analysis (Figure 2C). So, to confirm wheter the HMW-GSs are expressed in KK and JK transgenic rice lines, glutelin alcohol fractions were extracted from two Korean wheat cultivars, KeumKang and JoKyung, and the transgenic rice lines. Figure 3 showed that HMW-GSs consist of 1Ax2*, 1Dx5, 1Bx7, 1By8 and 1Dy10 in KeumKang and 1Ax1, 1Dx5, 1Bx7, 1By8, and 1Dy10 in JoKyung, as previously reported in [36]. The expression of wheat HMW-GSs, 1Dx5_KK, and 1Dy10_JK, was observed in KK and JK transgenic lines, respectively. Oszvald et al. [32,33] reported that the significant over-expression of 1Dx5 in rice seeds results in changing storage protein composition and increasing disulfide isomerase (PDI) levels, causing a positive effect on the functional properties of rice dough. Previous studies reported that the significant suppression of one SSP is compensated for by the increased accumulation of other SSPs, including PDI [1,40,41]. Takaiwa et al. [10] found that the expression of a foreign gene caused altered SSP levels in transgenic rice. However, in our study, we did not observe differential expression of endogenous SSPs between wild-type and transgenic rice lines in SDS-PAGE analysis, unlike our past report [41]. The maintenace of the wild-type SSP profile in transgenic KK and JK rice lines may be due to low transgene expression levels.

### 2.3. Accumulation of Wheat HMW-GSs in Transgenic Rice Endosperms

**Figure 4:**
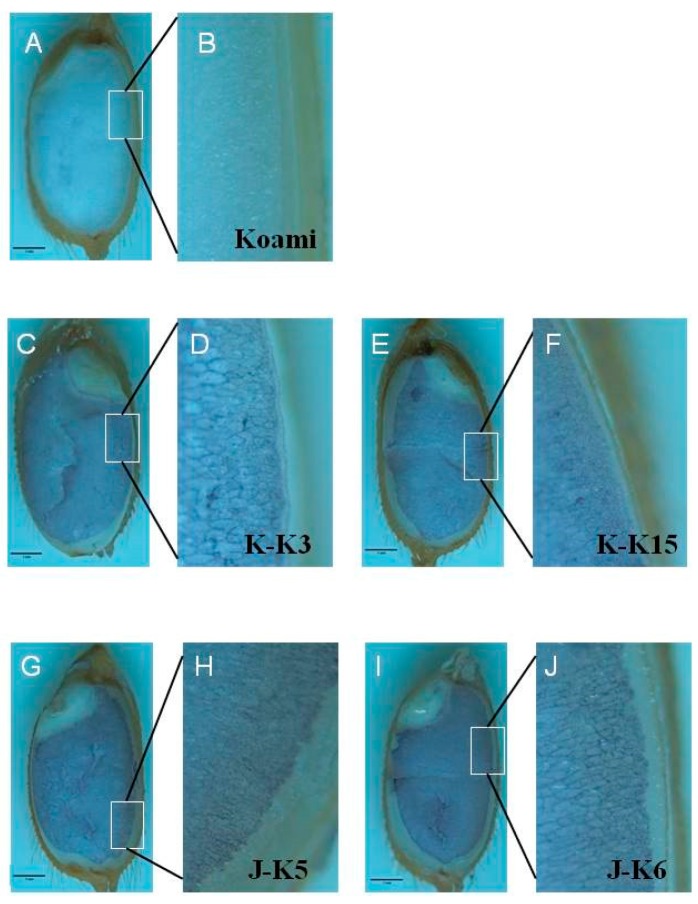
In situ immunohybridization in mature seeds of rice wild-type cultivar Koami (**A**,**B**) and independent transgenic rice lines overexpressing wheat HMW-GSs. KK-3 and KK-15 transgenic rice lines express HMK-GS 1Dx5_KK (**C**–**F**) and JK-5 and JK-6 lines express HMK-GS 1Dy10_JK (**G**–**J**). Each white box region in A, C, E, G and I is scaled up to B, D, F, H and J, respectively. Black scale bar: 1 mm.

To characterize 1Dx5_KK and 1Dy10_JK accumulation in transgenic rice seeds, we observed their expression in different seed developmental stages and morphologies. In transgenic rice seeds, wheat HMW-GS accumulation began 5–7 days after flowering (DAF) (Figure S2) and peaked at 15 DAF, similar to the expression pattern of rice glutelin. This indicated strict control of *1Dx5_KK* and *1Dy10_JK* expression by the *Glu-B1* promoter. Furthermore, in situ immunohybridization revealed that the 1Dx5_KK and 1Dy10_JK accumulated in the endosperm of transgenic rice seeds, predominantly in the aleurone layer than in the starchy endosperm (Figure 4). A similar accumulation pattern was reported in transgenic rice seeds overexpressing ferritin under control of the *Glu-B1* promoter [8]. In wheat seeds, HMW glutenin is concentrated in the starchy endosperm, which further indicates that HMW-GS accumulation in the aleurone layer of transgenic rice seeds is the result of *Glu-B1* promoter activity.

### 2.4. Cellular Localization of Wheat HMW-GSs in Transgenic Rice Seed Endosperm

**Figure 5:**
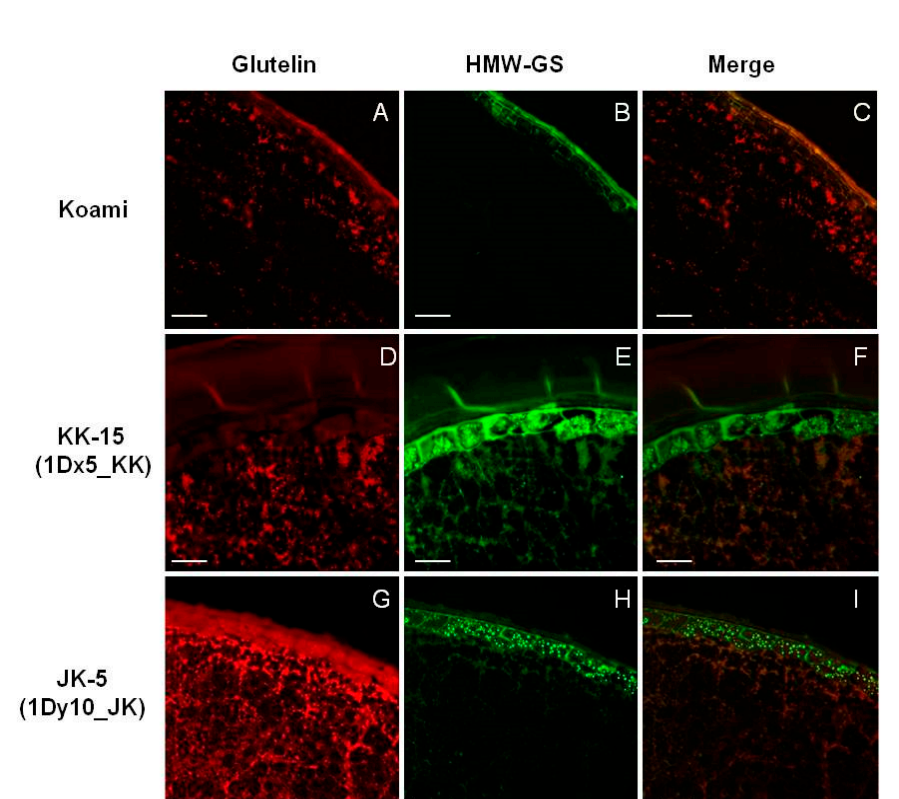
Immunofluorescence localization of glutelins and wheat high-molecular-weight glutenin subunits (HMW-GSs) in wild-type rice seeds of Koami (**A**–**C**) and transgenic rice lines KK-15 overexpressing 1Dx5_KK (**D**–**F**) and JK-5 overexpressing 1Dy10_JK (**G**–**I**). Fluorescence images were generated using red anti-glutelin antibodies (**A**,**D**,**G**) and green anti-HMW-GSs antibodies (**B**,**E**,**H**), which were then merged (**C**,**F**,**I**). White Scale bar: 100 μm.

**Figure 6:**
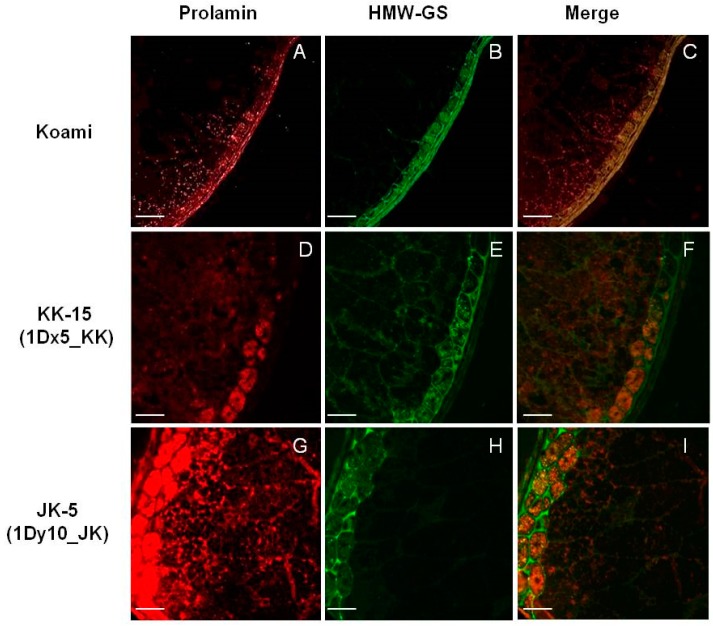
Immunofluorescence localization of prolamins and wheat HMW-GSs in wild-type rice seeds of Koami (**A**–**C**) and transgenic rice lines KK-15 overexpressing 1Dx5_KK (**D**–**F**) and JK-5 overexpressing 1Dy10_JK (**G**–**I**). Fluorescence images were generated using red anti-prolamin antibodies (**A**,**D**,**G**) and green anti-HMW-GSs antibodies (**B**,**E**,**H**); which were then merged (**C**,**F**,**I**). White Scale bar: 100 μm.

The major SSPs in rice are glutelin, prolamin, and globulin. Prolamin is deposited into ER-derived spherical protein bodies (protein body-I, PB-I), and glutelin and globulin are deposited into vacuole-derived protein bodies (protein body-II, PB-II) via the Golgi apparatus. To confirm the location of the wheat HMW-GSs in transgenic rice seeds, we performed immunofluorescence analysis using individual primary antibodies that are specific for glutelins, prolamins, and wheat HMW-GSs (Figure S3). In the seeds of wild-type rice cultivar Koami, the non-specific fluorescence signals of glutelins (red, Figure 5A), prolamins (red, Figure 6A), and wheat HMW-GSs (green, Figure 5B and Figure 6B) were observed in the outer aleurone layers, such as the tegmen, palea, and pericarp layers. The noise signals were subtracted by overlaying the fluorescence signals of glutelins and wheat HMW-GSs (orange, Figure 5C), and of prolamins and wheat HMW-GSs (orange, Figure 6C). In transgenic rice seeds expressing wheat HMW-GSs, namely the KK-15 line overexpressing 1Dx5_KK (Figure 5D–F) and the JK-5 line overexpressing 1Dy10_JK (Figure 5G–I), an overlap of the specific fluorescence signals for glutelins (red) and wheat HMW-GSs (green) was observed in the endosperm but not in the aleurone layers (Figure 5F,I). No fluorescence signal overlap was observed for prolamins and wheat HMW-GSs (Figure 6D–I), indicating that 1Dx5_KK and 1Dy10_JK do not accumulate in PB-I alongside prolamins. Moreover, the presence of both overlapping and distinct fluorescent signals for glutelins and wheat HMW-GSs in the transgenic rice seeds suggests that the wheat HMW-GSs accumulate simultaneously in PSVs (PB-II) and a novel type of protein body. Similar results were reported in transgenic rice seeds expressing wheat HMW-GS 1Dx5 [33]. Other studies have found that the expression of foreign proteins in rice seeds causes the formation of novel types of protein bodies and affects the cellular accumulation of rice SSPs [42,43,44,45].

## 3. Materials and Methods

### 3.1. Vector Construction and Rice Transformation

Wheat high-molecular-weight glutenin subunits (HMW-GSs) *1Dx5* (GenBank accession No. AB485591) and *1Dy10* (No. AB281268) genes encoding HMW-GS 1Dx5 (90.34 kDa) and 1Dy10 (69.7 kDa), respectively, were cloned from Korean wheat (*Triticum aestivum* L.) cv. KeumKang and cv. JoKyung using gene-specific primers, as listed in Table S1. The individual genes were inserted into pMJ103 destination binary vector using the Gateway cloning system [46]. The binary vector includes the rice seed endosperm-specific *Glu-B1* promoter, the *nopaline synthase* (*Nos*) terminator, and the *bialaphos resistance* (*Bar*) gene as an herbicide-resistance marker (Figure 2). The constructed binary vectors were introduced into *Agrobacterium tumefaciens* (LBA4404) and then the genes of interest were inserted into the genome of *japonica*-type Korean rice cultivar Koami, as previously described in [47]. Wild-type cv. Koami and transgenic rice plants were cultivated in the GMO (Genetically Modified Organism) field of the National Institute of Agricultural Sciences and the harvested seeds were stored in a 4 °C cold room.

### 3.2. Antibodies

Polyclonal-rabbit antibody specific to rice glutelin B subunit (polyclonal-rabbit anti-GluB) was prepared previously in our laboratory [41]. Wheat SSPs were extracted from the flour of JoKyong (*Triticum aestivum* L.) and HMW-GSs were separated by SDS-PAGE. Polyclonal-rat antibody that is specific to wheat HMW-GS (polyclonal-rabbit anti-HMW-GS) was produced using the separated HMW-GSs. Moreover, polyclonal-rabbit antibody that is specific to 13-kDa prolamin was received from professor Ahn, Bong-Whan (Chonnam National University, Gwangju, Korea).

### 3.3. Quantitative Real Time-Polymerase Chain Reaction (qRT-PCR)

Mature dry seeds of wild-type and independent transgenic rice lines were ground to a fine powder using a mortar and pestle. Powdered samples (100 mg) were used for total RNA extraction according to the CTAB (cetyl trimethyl ammonium bromide) extraction method, coupled with an RNA Clean-up using RNeasy Plant Mini Kit (QIAGEN, Germantown, MD, USA), as previously described [48]. The quality, yield, and purity of RNA were determined using a NanoDrop 1000 spectrophotometer (Thermo Scientific, Waltham, MA, USA). Total RNA samples were first DNase-treated with RNase-free DNase (Stratagene, Agilent Technologies, La Jolla, CA, USA), and then first-strand cDNA was synthesized in a 20-μL reaction mixture using a SuperScript III First-Strand Synthesis System Kit (Invitrogen, Carlsbad, CA, USA) and 1 μg of total RNA, according to the manufacturer’s procedure. Thermal-cycling parameters of the CFX96^®^ Real-Time Detection System (Bio-Rad, Hercules, CA, USA) were as follows: an initial denaturation at 95 °C for 15 min, a cycling regime of 40 cycles at 95 °C for 10 s, 55 °C for 10 s, and 72 °C for 30 s, and then a 65–95 °C gradient to produce the melting curve. The primer sequences used for the qRT-PCR are listed in Table S1. Total RNA extractions from seeds of wild-type cv. Koami and transformants were performed from three independent experiments and the expression value of each gene was represented by the mean of three independent biological replicates.

### 3.4. SDS-PAGE and Immunoblotting

To extract total SSPs, rice flour (100 mg) was agitated with 1 mL of SDS-urea buffer (4% SDS, 8 M urea, 0.25 M Tris-HCl pH 6.8, 20% glycerol, 0.01% bromophenol blue, and 5% β-mercaptoethanol) for 3 h at room temperature, and then centrifuged at 14,000× *g* for 10 min. The glutenin fraction from rice flour (20 mg) was extracted using 55% 1-propanol, as described previously [49]. The extracted total SSPs (10 μg) and gliadin fraction were all separated on 12.5% SDS-PAGE gels, stained using Coomassie brilliant blue (CBB) staining solution (0.1% (*w*/*v*) CBB R-250, 45% (*v*/*v*) methanol, and 45% (*v*/*v*) glacial acetic acid) for 3 h, and then destained in 10% methanol, 10% glacial acetic acid, and 80% distilled H_2_O. Furthermore, the separated proteins on gels were blotted onto polyvinylidene difluoride membranes (PVDF; Bio-Rad) using a semi-dry transfer machine (Bio-Rad) for 2 h. Immunoblot analysis was performed using polyclonal-rat anti-HMW-GS primary antibody (diluted 1:2000) and horseradish-peroxidase (HRP)-conjugated anti-rat IgG secondary antibody (1:10,000, Promega, Madison, WI, USA). Signals of target proteins were visualized using a luminescence image analyzer (Chemiluminesence Fusion SL, Vilber Lourmat, Marne-la-Vallée, France).

### 3.5. In Situ Immunohybridization

Mature seeds were soaked in distilled water overnight at 4 °C and were sectioned lengthwise using razor blades. The seed sections were washed in distilled water, incubated in blocking solution (TBS, pH 6.8 containing 2% (*w*/*v*) skimmed milk) for 3 h, and then incubated overnight at 4 °C in blocking solution with polyclonal-rat anti-HMW-GS primary antibody (1:2000). Sections were washed three times with TBS containing 0.05% Tween-20 before the addition of alkaline phosphatase (AP)-conjugated anti-rat IgG secondary antibody (1:10,000, Promega), followed by a further three washes in TBS with 0.05% Tween-20. Target proteins were visualized using ProtoBlot^®^II AP system (Promega) and observed using a stereoscopic microscope (Leica M205C Microsystem, Leica, Heerbrugg, Switzerland).

### 3.6. Immunofluorescence

Mature rice seeds were embedded with LR white resin (London Resin, Hampshire, UK), according to Satto’s method [21]. 1 µm thick sections for microscopy were prepared using an ultramicrotome that ws equipped with glass knife (Leica) and mounted onto slides. The specimens on slides were incubated in PBS containing 0.1 M NH_4_Cl for 5 min, blocked for 30 min with 5% bovine serum albumin (BSA) in PBS, and then incubated for 2 h with a mixture of two primary antibodies (1:2000), such as polyclonal-rabbit anti-glutelin/polyclonal-rat anti-HMW-GS and polyclonal-rabbit anti-prolamin/polyclonal-rat anti-HMW-GS. Following primary antibody incubation, the specimens were rinsed with 0.1% BSA in PBS for 10 min and incubated in PBS containing 5% BSA and fluorescence-labeled secondary antibodies (1:1000) in the dark, such as anti-rabbit Arexa fluor^®^ 594 (Invitrogen, Eugene, OR, USA) conjugated and anti-rat Arexa fluor^®^ 488 (Invitrogen) conjugated. The specimens were washed with PBS for 10 min and were transferred to mounting solution (VECTASHIELD^®^ (Vector Laboratories, Burlingame, CA)) Hard*Set™Antifade Mounting Medium). The incubated specimens were then analyzed using an Axioplan 2 imaging microscope (Carl Zeiss, New York, NY, USA).

## 4. Conclusions

With the aim of improving the processing suitability of rice flour through the transgenic expression of viscoelastic proteins that confer high dough elasticity, we described here the cloning of wheat HMW-GSs *1Dx5_KK* and *1Dy10_JK* from Korean wheat cultivars KeumKang and JoKyung, respectively, and their subsequent overexpression in transgenic rice seeds. In situ immunohybridization demonstrated that the 1Dx5_KK and 1Dy10_JK accumulated within the endosperms of transgenic rice seeds. Furthermore, immunofluorescence localization of these wheat HMW-GSs indicated that they were deposited into protein storage vacuoles (PSVs, PB-II) alongside rice glutelins and into a novel protein body type via a currently unknown mechanism. This work describes fundamental results that improve our understanding of the interaction between endogenous and engineered SSPs in rice, which is an important step for future plant engineering.

## Supplementary Materials

Supplementary materials can be found at www.mdpi.com/1422-0067/18/11/2458/s1.
