# Supplementary material for: Cellular Localization of Wheat High Molecular Weight Glutenin Subunits in Transgenic Rice Grain
Source: Int J Mol Sci. 2017 Nov 18;18(11):2458. doi: 10.3390/ijms18112458 (PMC5713424; doi:10.3390/ijms18112458)
Supplement: Supplementary file 1 [file ijms-18-02458-s001.pdf]

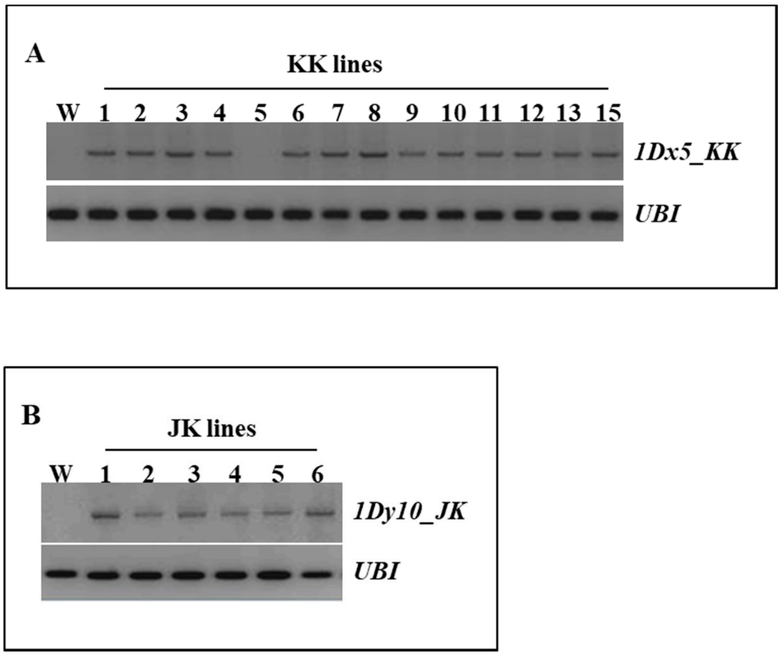

Figure S1. Reverse transcription polymerase chain reaction (RT-PCR) analysis of wheat HMW-GSs, *1Dx5_KK* and *1Dy10_JK* in Koami (wild-type, W) and independent transgenic rice lines. (**A**) KK lines (15) overexpressing 1Dx5_KK ; (**B**) JK lines (6) overexpressing 1Dy10_JK; UBI, ubiquitin

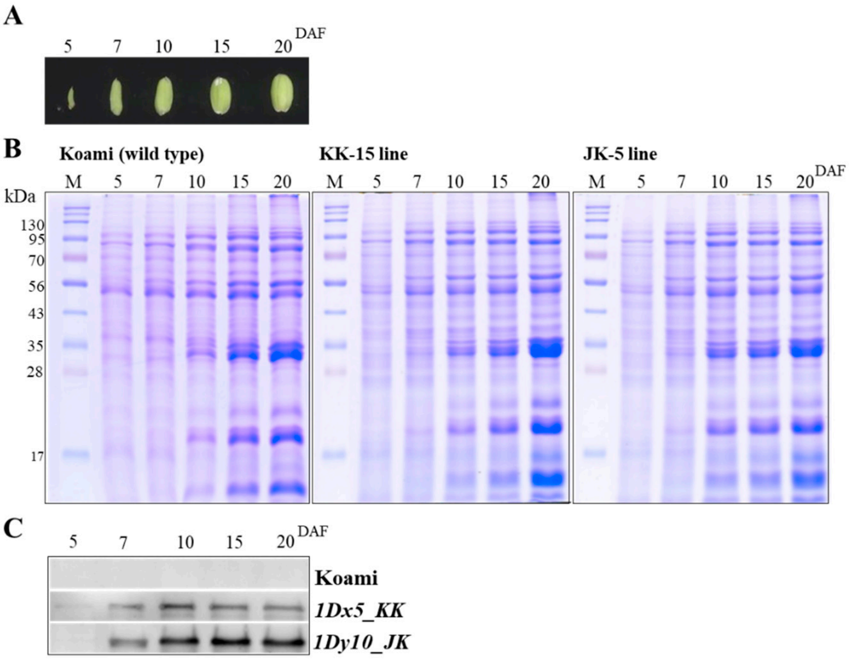

Figure S2. Molecular events according to see development stage in Koami (wild-type), KK-15 line overexpressing 1Dx5_KK and JK-5 line overexpressing 1Dy10_JK. **A**) Morphological changes according to seed maturation; **B**) Expression analysis of seed storage proteins at 5-20 day after flowering (DAF) using SDS-PAGE; **C**) Expression analysis of wheat HMW-GSs at 5-20 DAF using western blot.

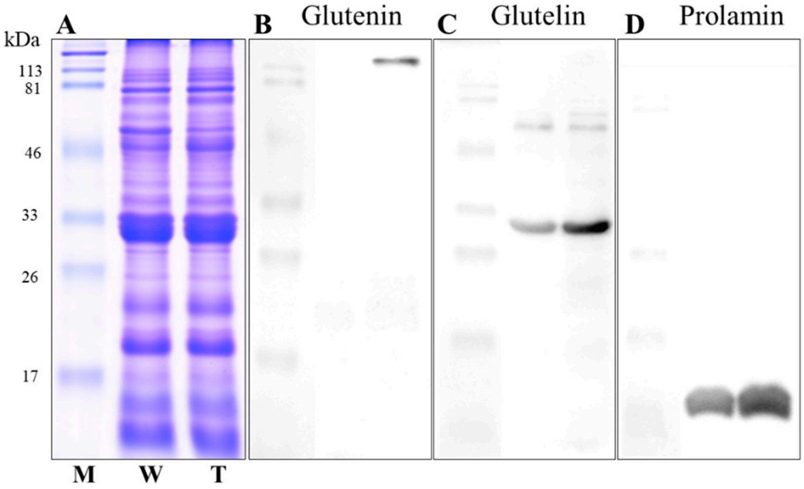

Figure S3. Specificity Analysis of three primary antibodies to glutenin, glutelin and prolamin for immunofluorescence. (**A**) The separation of total seed storage proteins extracted from Koami (wild type, W) and transgenic rice (T, KK-3 line) using SDS-PAGE. Western blot analysis with polyclonal-rat anti HMW-glutenin (**B**), polyclonal-rabbit anti glutelin B1-2 subunit (**C**) and polyclonal-rabbit anti 13kD prolamin (**D**). M, marker.

Table S1. Sequence of primers used for cloning and qRT-PCR.

Gene symbol	Target gene	Accession #	Forward primer	Reverse primer	Product size
1Dx5	HMW-GS *Glu1Dx5*	AB485591	5′-AAAAAGCAGGCTATGGCTAA GCGGTTAGTCCTCTTTGTG-3′	5′-AGAAAGCTGGGTCTATCACTGG CTCGACAATGCGTC-3′	2547
1Dy10	HMW-GS *Glu1Dy10*	AB281268	5′-AAAAAGCAGGCTATGGCTAA GCGGCTGGTCCTCTTTGCG-3′	5′-AGAAAGCTGGGTCTATCACTGG CTAGCCGACAATGCGTC-3′	1947
1Dx5-RT	HMW-GS *Glu1Dx5*	AB485591	5′-CAACAGCTCCGAGACATTAG-3′	5′-GTGCAGGTATTCCCCAAAAT-3′	169
1Dy10-RT	HMW-GS *Glu1Dy10*	AB281268	5′-ACTGCAGCAACTCCAACAAG-3′	5′-GGAGAAGCTTGGCCTGGATA-3′	117
UBI	*Ubiquitin*	-	5′-TGGTCAGTAATCAGCCAGTTT GG-3′	5′-GCACCACAAATACTTGACGAA CAG-3′	81
